# A chromosome-scale reference genome for *Giardia intestinalis* WB

**DOI:** 10.1038/s41597-020-0377-y

**Published:** 2020-02-04

**Authors:** Feifei Xu, Aaron Jex, Staffan G. Svärd

**Affiliations:** 1 Department of Cell and Molecular Biology, BMC, Box 596, Uppsala University, SE-751 24 Uppsala, Sweden; 2 Population Health and Immunity Division, Walter and Eliza Hall Institute of Medical Research, Parkville, Victoria, 3052 Australia; 3 Faculty of Veterinary and Agricultural Sciences, The University of Melbourne, Parkville, Victoria 3010 Australia

**Keywords:** Genome, Comparative genomics, Pathogens, DNA sequencing, Parasitic infection

## Abstract

*Giardia intestinalis* is a protist causing diarrhea in humans. The first *G. intestinalis* genome, from the WB isolate, was published more than ten years ago, and has been widely used as the reference genome for *Giardia* research. However, the genome is fragmented, thus hindering research at the chromosomal level. We re-sequenced the *Giardia* genome with Pacbio long-read sequencing technology and obtained a new reference genome, which was assembled into near-complete chromosomes with only four internal gaps at long repeats. This new genome is not only more complete but also better annotated at both structural and functional levels, providing more details about gene families, gene organizations and chromosomal structure. This near-complete reference genome will be a valuable resource for the *Giardia* community and protist research. It also showcases how a fragmented genome can be improved with long-read sequencing technology completed with optical maps.

## Abstract (summary)

**Table:** 

Measurement(s)	DNA • sequence_assembly • sequence feature annotation
Technology Type(s)	DNA sequencing • sequence assembly process • sequence annotation
Sample Characteristic - Organism	Giardia intestinalis

Machine-accessible metadata file describing the reported data: 10.6084/m9.figshare.11695659

## Background & Summary

*Giardia intestinalis* has a duplicated cell structure with two transcriptionally active nuclei. It is one of the most prevalent parasitic protists causing diarrhea, infecting a wide range of hosts, including humans. *Giardia* has been categorized into eight different assemblages (genotypes, A-H) based on host-specificity and genetic differences^1^. Assemblage A and B infect humans, with WB isolate from assemblage A being the most extensively studied. The first sequenced *G. intestinalis* genome is from the WB isolate, and was published in 2007^2^. At the time, 200,000 reads from both ends of small-insert plasmid libraries and 2,400 end sequences from a 200 kbp library in bacterial artificial chromosome (BAC) vectors were generated and sequenced with Sanger sequencing. This yielded a genome of 11.7 Mbp in size, distributed on 306 contigs (92 scaffolds)^2^ (Table 1). Since then, this genome has been used as the reference genome for *Giardia* research. Subsequently, a few other *Giardia* genomes have been published, including human isolates (GS, GS-B and BAH15c1) from assemblage B^3–5^, human isolates (DH, AS175 and AS98) from assemblage A2^4,6^, one pig isolate (P15) from assemblage E^7^ and two dog isolates from assemblages C and D^8^. The availability of several genomes has advanced *Giardia* research and it is now possible to use different omics techniques during studies *in vitro* and *in vivo*.

**Table 1:** Comparison of the old and the new *G. intestinalis* WB genome.

	Old	New
Sequencing instrument	LI-COR, ABI 3700	PacBio RS II
# Reads	224,000*	411,835
# Bases	NA	3.6 billion
Coverage	11×	200×
Assembler	ARACHNE 2.0	HGAP3
Optical mapping	−	+
# Chromosomes	5	5
Genome size (Mbp)	11.7	12.6
# Contigs	306	38
# Scaffolds	92	35
# Gaps	137	4
Gap size (Mbp)	1.6	0.9
G + C %	49.0	46.3
ASH %	0.01	0.03
# Genes	5,901	4,963
Mean gene length (aa)	530	635
Coding density %	81.6	81.5
Mean intergenic region (bp)	481	477
Number of introns**	4	8 cis, 5 trans
tRNAs	63	65
5S rRNAs	8	10
5.8S rRNAs	1	10
18S rRNAs	4	9 (2 partial)
28S rRNAs	4	4 (5 partial, 12 ψ)

*200,000 from end sequences with small insert, 2,400 end sequences from 200 kbp long insert.

**There were 4 identified intron-containing genes in the first published draft genome. Not all the discovered intron-containing genes were properly integrated in the newest GiardiaDB either. This version integrates all the discovered introns, which is consistent with our results searching for the *de novo* introns.

As part of diplomonads, a group of unicellular protists, *Giardia* genomes have made it possible to perform comparative genomic studies within diplomonads. Comparative genomics between the genomes of the salmon parasite *Spironucleus salmonicida* and *Giardia* revealed how *S. salmonicida* adapted to colonize different sites in the host^9^. Comparative studies between the transcriptome of free-living diplomonad *Trepomonas* and parasitic diplomonad genomes revealed how a free-living organism evolved from its parasitic ancestor^10^.

However, all published *Giardia* genomes are fragmented, due to the limitation of the sequencing technologies used at the time. Fragmented genomes have limited our understanding on chromosome structure and how the genome evolves at the chromosomal level. During the last years, long-read third generation sequencing has matured. For example, Pacbio claims that its newest HiFi sequencing can generate reads up to 300 kbp with half of the bases in reads >160 kbp^11^.

To obtain a high-quality reference genome of *G. intestinalis*, we have re-sequenced the WB genome using Pacbio technology. Long-reads were assembled into long contigs, which were then scaffolded into near-complete five chromosomes with the aid of optical maps^12^. Using a comparative genomics approach and RNA-Seq reads mapping, we could also improve both the structural and functional annotations.

## Methods

### DNA preparation and sequencing

*G. intestinalis* WB/C6 (ATCC 50803) was cultivated according to ref. ^13^ in TYDK media in tightly capped slanted culture tubes at 37 °C. Total genomic DNA was extracted from 2.3 × 10^8^ trophozoites using Qiagen Blood & Tissue Kit 50 (per manufacturer’s instructions), and purified using phenol-chloroform extraction. It was then further purified with Qiagen Genomic-tip 20/G. The concentration and quality of the extracted DNA was determined by NanoDrop and agrose gel electrophoresis. Genomic DNA (5.6 *μ*g) was sequenced with PacBio RS II using 8 SMRT cells at the Uppsala Genome Center at the Science for Life Laboratory (Uppsala University), which generated 411,835 reads in 3.6 billion bases (Table 1). Reads have an N50 length of 12.7 kbp and 10% them are longer than 20 kbp.

To assist base error correction, the PacBIO long-read data was supplemented with short-read (Illumina) sequence data at a high coverage (100X). These short-read data was generated from *G. intestinalis* WB/C6 grown in TYDK media as per^13^ in a 25 mL tissue culture flask. Total genomic DNA was extracted from 1 × 10^7^ trophozoites using the Qiagen Powersoil Kit (per the manufacturer’s instructions). Approximately 1 *μ*g of genomic DNA was prepared for Illumina Truseq library construction (fragment size 300 bp) and subjected to paired-end (75 bp) sequencing on Illumina HiSeq 2000 per the manufacturer’s instructions. This generated 16 million paired-end reads.

### Genome assembly

**Fig. 1:**
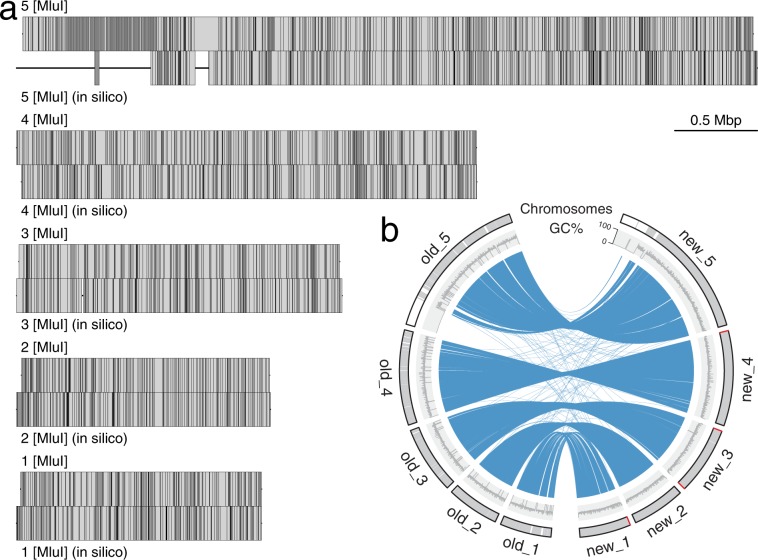
Near-complete five chromosomes. (**a**) Restriction enzyme (MluI) maps of the five chromosomes aligned with the genomic sequences digested with MluI *in silico*. Each vertical line inside boxes represents a restriction enzyme cutting site. Gaps in the genomic sequences are represented with a horizontal line outside of boxes. (**b**) Circular plot comparing the old five chromosomes (to left) to the new five chromosomes (to right). Chromosomal sequences are represented in grey at the outermost circle with gaps in white bands and telomeric repeats in red. BLASTN matches between the two genomes are shown as blue ribbons in the middle. R package circlize (v0.4.8) was used to draw the circular plot^39^.

SMRT Analysis (v2.3.0) pipeline^14^ provided by Pacbio was used for the Pacbio reads. Software mentioned below without version information were all from the SMRT Analysis pipeline. Reads were assembled *de novo* with HGAP^14^ followed by consensus sequence calling with Quiver^14^. This yielded 80 contigs. Those contigs were then mapped to the optical maps of the five chromosomes (Fig. 1a)^12^ using MapSolver (v3.2.0) provided by OpGen. Neighboring contigs were then stitched together as follows: If overlaps were identified between the neighboring contigs, the two contigs were merged based on the aligned overlapping sequence; If no overlap was detected, the two contigs were stitched with Ns in between to represent gap with size determined by the aligned optical map. The resulting five scaffolds were fed into BridgeMapper workflow^14^, which produces split alignments using BLASR. The split alignments were visualized using SMRT View, and no misassemblies and structural variation were detected. PBJelly (v15.8.24)^15^ was used to further close the gaps in the five scaffolds using the Pacbio reads. The scaffolds were then further polished with Quiver^14^, and the reads failed to map to the scaffolds were assembled independently with canu (v1.4)^16^. Canu contigs and the five scaffolds were combined, and further polished with Quiver. This resulted in 35 scaffolds, with the five major ones representing 97% of the total size at 12.6 Mbp, slightly bigger than the old draft genome (Table 1). The five major scaffolds representing the five *Giardia* chromosomes contain only four internal gaps compared to the 137 gaps in the old version (Table 1, Fig. 1b).

During the annotation stage below, we noticed that some full length genes were truncated in the new draft genome due to frame-shifts caused by homopolymer errors introduced by PacBio reads. Illumina DNA sequencing reads were mapped to the draft reference using BWA (v0.7.15) short read alignment component^17^ and the alignment bam file was sorted by samtools (v1.8)^18^ and its mpileup function was used to generate pileup file listing per base mapping information. Pileup file was parsed and indels and SNPs were updated when the alternative base/indel has at least 50% support from at least 10 reads mapped. This resulted in 50 SNPs updated affecting 20 genes, and 85 indels updated affecting 38 genes.

### Heterozygosity estimation

Illumina reads were re-aligned to the base-corrected reference genome using BWA (v0.7.15) and samtools (v1.8) mpileup result was re-generated. SNP (or allelic sequence heterozygosity (ASH)) sites mentioned below were called in positions of at least 20X base coverage with an alternative base in at least 10% of the reads.

### Genome annotation

The previously published *G. intestinalis* WB genome was downloaded from GiardiaDB (v36.0)^19^ and the annotation was transferred to the new reference genome using RATT (v0.95)^20^. GlimmerHMM (v3.0.1)^21^ and Prodigal (v2.6.3)^22^ were used for *de novo* gene prediction, and 500 RATT transferred annotations were selected to train GlimmerHMM prediction. Similarity information from BLASTP^23^ against NR database and domain information from Conserved Domain (CD) search^24^ were combined for functional annotation. RATT transferred annotations and *de novo* predictions were merged, and the ones inconsistent of the three sources were manually examined. RNA-Seq reads (SRR10063826^25^) were mapped to the genome with BWA (v0.7.15), and the mapping information were used as a guideline for structural annotation. Manually curated annotations provided by *Giardia* researchers^19^ were also incorporated into the functional annotation. Inconsistent annotations between the old version and the new version were double checked to ensure the updated annotation was an improvement. For multi-copy genes, synteny between the two versions were examined to determine the most appropriate geneid for the gene, an attempt to keep the synteny consistent between the two versions of genomes.

Introns in *Giardia* have been published in different papers^2,26,27^, but were not curated and integrated in the genome. In this version of annotation, we integrated all the 8 cis-spliced introns and 5 trans-spliced introns (Table 1). Searching for the new general intron motif did not reveal new intron candidates.

We have in total annotated 4,963 protein-coding genes plus an additional 306 pseudo genes in the new genome (Table 1). Although the number of genes is reduced compared to the old genome, the new genome has on average longer genes and smaller intergenic regions (Table 1). The coding percentage remains the same.

**Fig. 2:**
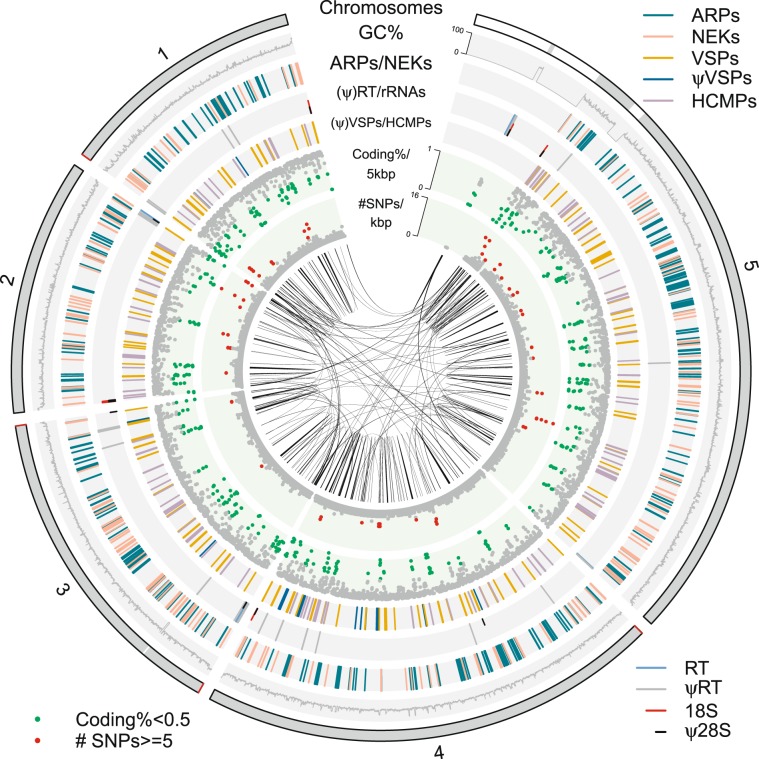
Circular plot of the five chromosomes. Chromosomal sequences are represented in grey at the outermost circle with gaps in white bands and telomeres in red. Inner tracks are arranged as: GC%, ARPs/NEKs, (ψ)Reverse transcriptases/rRNAs, (ψ)VSPs/HCMPs, Coding density, SNPs density, regions with similarity. Regions with similarity represent BLASTN matches against itself with >95% sequence identity and >2000 bp in length. The circular plot was drawn with R package circlize (v0.4.8)^39^.

18S, 28S and 5S ribosomal RNAs (rRNAs) were predicted using rnammer (v1.2)^28^. 5.8S rRNA sequence was retrieved from NCBI, then searched against the new genome with BLASTN to locate all the copies. tRNAs were identified using tRNAscan-SE (v1.23)^29^. There are more duplicated copies of tRNAs and rRNAs in the new genome (Table 1). 5 S rRNAs are found internally, while 18S, 5.8S and 28S rRNAs are found at most of the chromosome ends (Fig. 2). Two partial 18S rRNAs sit at the two ends of the biggest internal gap at the 5′ end of chromosome 5 (Fig. 2), which suggest the unfilled gap could be caused by tandem copies of rRNAs. If so, it will make this ribosomal RNAs (28S + 5.8S + 18S) region 318 kbp long, approximately 86 copies of rRNAs.

### Gene family annotation and distribution

*Giardia* has a large group of cysteine-rich proteins, which are divided into 3 subgroups, including variant surface proteins (VSPs), high cysteine membrane proteins (HCMPs) and high cysteine proteins (HCPs). VSPs are secreted to the membrane and thus carry 5′ signal peptide and have a defined 3′ tail with a transmembrane domain followed by CRGKA pentapeptide motif. VSPs without proper signal peptide predicted by signalP (v5.0b)^30^ were either adjusted to the right start codon, if possible, or annotated as pseudoVSPs (pVSP, ψVSP). On top of lacking proper signal peptide, ψVSPs also tend to sit in arrays without proper start codons. In the end, we have 133 VSPs compared to 196 in the old genome, and an additional 208 ψVSPs.

Among the 133 VSPs, there are 38 in duplicated pairs. 12 pairs have a → ← orientation, and 7 pairs have a ← → orientation. Among the 7 pairs pointing away from each other, 4 pairs contain a reverse transcriptase remnant in between, and one contains a NEK kinase in between (Table 2).

**Table 2:** Arrangement of VSP pairs.

Chr	Gene1	Gene2	Arrangement	Gene size (aa)	Distance (bp)	Genes between
5	14586	d14586	→ ←	719	2689	
5	137722	137723	← →	661	2513	
5	137714	11470	→ ←	627	2669	
5	137708	137707	→ ←	593	2777	
5	d14331	14331	→ ←	419	2637	
4	16501	d16501	→ ←	692	2526	
4	d103992	103992	→ ←	627	2758	
4	50229	d50229	← →	740	3242	ψRT
4	d112801	112801	← →	747	6264	ψRT
4	111933	111936	← →	741	3846	ψRT
3	119706	119707	← →	673	3301	ψRT
3	115830	115831	→ ←	633	2813	
3	136003	136004	→ ←	551	2686	
2	d11521	11521	→ ←	628	2747	
2	d117204	117204	→ ←	255	2720	
2	117472	117473	← →	200	3101	NEK
2	50359	134710	← →	636	12440	
1	d115797	115797	→ ←	682	2649	
1	d112208	112208	→ ←	596	2648	

NEK kinase and the arbitrarily named protein 21.1 share ankyrin-repeat domains, and there are 179 NEK kinases and 242 protein 21.1 in the old genome. In the new genome, ankyrin-repeat containing genes are named as follows: Proteins with both kinase domain and ankyrin-repeat are called NEK kinases, and there are 184 of them; Proteins with only ankyrin repeats are called ankyrin repeat protein 1 (ARP-1), and there are 267 (+5 pseudo copies) of them; Proteins with ankyrin repeat as well as zinc-finger domain(s) are called ankyrin repeat protein 2 (ARP-2), and there are 33 of them; Proteins with ankyrin repeat as well as domain(s) other than a zinc-finger domain are called ankyrin repeat protein 3 (ARP-3), and there are 5 of them. The domains were found using HMMER3 (v3.0)^31^ search against Pfam (v31.0)^32^.

It was already observed, while comparing genomes from different *Giardia* isolates, that conserved synteny in *Giardia* genomes breaks at regions enriched with multi-gene families including cysteine-rich protein families, NEKs and ARPs^3^. With this near-complete genome, we could see how these gene families are distributed along the chromosomes (Fig. 2). The gene families are all over the chromosomes. *Giardia* genome is packed with genes with 81% genome coding and small intergenic region. However, regions around VSPs tends to be gene-poor (Fig. 2, green dots in coding% track), and those regions also have higher allelic sequence variation in the mapped reads (Fig. 2, red dots in SNPs track).

## Data Records

This Whole Genome Shotgun project has been deposited at DDBJ/ENA/GenBank under the accession AACB00000000. The version described in this paper is version AACB03000000^33^, and the GenBank assembly accession is GCA_000002435.2^34^. To distinguish the duplicated gene from the original one, ‘d’ was added after locus_tag GL50803_; To distinguish this version of annotation from the old one, ‘00’ was added after locus_tag GL50803_ in all the new geneids. The datasets generated and analyzed in this project are also available via GiardiaDB. Raw DNA sequence reads from Pacbio and Illumina are deposited at NCBI Sequence Read Archive (SRA) under accession number SRP191500^35^.

## Technical Validation

### Genome completeness

The five assembled chromosomes correlate well with the optical maps (Fig. 1a), with 92.2% of the optical maps covered by the assembled sequence. The biggest missing pieces lie at the 5′ end of the chromosome 5 (Fig. 1). From the sequences mapped to that region, we could see approximately four copies of non-LTR LINE-like retrotransposon GilM (Fig. 2)^36^. One copy of GilM is 5,474 bp long and encodes a reverse transcriptase (RT). Mapping all the raw reads to the region gives an average coverage at 1,800X. While the whole genome was sequenced at 200X coverage, it indicates that there are at least 9 copies of this GilM in the genome. The size and the repetitive pattern of the optical map at the 5′ end of the chromosome 5 (Fig. 1a) indicate more than 9 copies of GilM. This difference could be caused by subtelomeric size variation, which has been widely observed in humans and yeast^37,38^, and it has been observed that the chromosome sizes vary in A isolates^6^. Besides that, the chromosomal ends could have been sequenced at less coverage than average.

Synteny between the old and the new genome is highly conserved, with the new genome being more complete. Four out of ten chromosome ends were assembled into telomeric repeats (TAGGG)n (Fig. 1b). The orientation of the chromosomal sequences in the new genome is ordered after the orientation of the optical maps, which is reverse complement to the old one (Fig. 1b).

The remaining 33 contigs are all small with sizes <33 kbp, and enriched with repeats, ribosomal RNAs and gene families.

The new genome is also more complete in reconstructing duplicated regions. In fact 6.9% of the genome resulted from duplications. Duplications are more commonly observed in short stretches of DNAs with just one gene, but multi-gene large duplications up to 20 kbp are also observed. This might be the largest duplication the assembly could resolve due to the limitation of read length. Members of multi-gene families are often seen in the duplicated regions.

### Annotation improvement

**Fig. 3:**
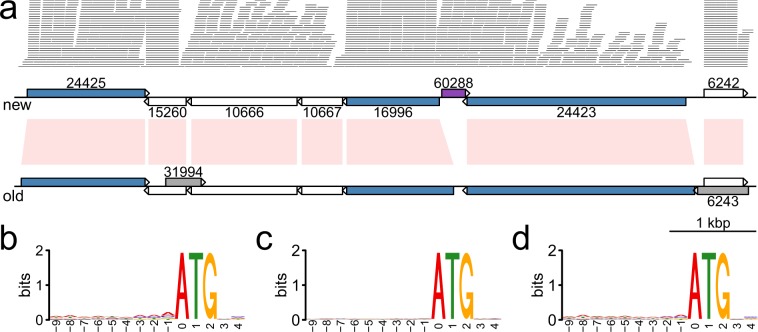
An 8 kbp genomic region on chromosome 5 with improved annotation and start codon sequence logos. (**a**) An 8 kbp long genomic region located on chromosome 5. The first part shows the RNA-Seq reads mapped to the region. A coverage cutoff 30 was used for better display. The new genome is drawn directly below the RNA-Seq reads with the old genome aligned at the bottom. Orthologs between the two genomes are linked with light pink bands. Genes are shown in arrowed boxes filled with different colors. White indicates genes without modification, purple (60288) indicates the new BolA-like gene to the new genome, grey represents the unique genes to the old genome (deleted from the new genome). Blue represents genes with adjusted start codons, and in these three genes, they were shortened with updated descriptions. (**b**) Sequence logo at the start codon of the 626 new genes with updated start codons. (**c**) Sequence logo at the start codon of the 626 old genes from the old genome. (**d**) Sequence logo at the start codon of all the 4,963 protein-coding genes. Sequence logos were drawn with R package seqLogo (v1.50)^40^.

The first published *Giardia* WB genome was over-annotated with small open reading frames (ORFs) overlapping real genes, as well as genes with wrongly assigned start codons (Fig. 3a). This is problematic for many analyses like unique genes, intergenic regions and regulatory elements. We used information from domain searches, start codon motifs, RNA-Seq read alignments and homologs from other *Giardia* genomes, to clean up and improve the structural and functional annotations.

Among the 4,963 protein-coding genes, 2,948 genes stay exactly the same, 17 stay the same but with SNPs, 1,173 genes have the same sequences but updated descriptions. 626 genes have adjusted start codons, based on the alignments of orthologous genes within *Giardia* as well as the motifs around the start codons. In fact, the new start codons often sit after a stretch of A-rich sequences (Fig. 3b), while this motif is lacking at the start codons of the old genes (Fig. 3c). This A-rich motif is also inline with the general sequence profile at the start codons for the whole genome (Fig. 3d)^2^. 55 genes are new to the new genome, and most of their sequences were found in the old genome but not annotated due to misannotations. 138 genes are new to the new genome but share sequence similarity with genes already annotated. 6 genes are the same but have part of the sequences changed due to frame-shifts in the new reference genome.

Figure 3 showcases an 8 kbp region on chromosome 5 where the new genome has better annotation. In this 8-gene region, 3 of the genes were shortened with now the start codons better matching the RNA-Seq reads mapped (Fig. 3a), as well as the genes start with A-rich motif right next to or close to the start codons (Fig. 3b). All these three genes got updated functional annotations including domain information. Shortening GL50803_16996 also allowed space for a new gene GL50803_0060288, BolA-like protein. The two grey colored hypothetical genes in the old genome, overlapping completely with the functional genes, are most likely misannotations and were thus removed in the new genome.

The new genome has also improved annotation on hypothetical proteins with now 2,099 hypothetical proteins instead of 3,545 in the old genome. 955 hypothetical proteins in the old genome were removed in the new genome due to reasons stated above, and 69 hypothetical proteins are new to the new genome. 598 hypothetical proteins have new functional annotation based on domain and BLAST matches, and 38 functionally annotated genes in the old genome were changed to hypothetical proteins due to lack of support for the functions.

## Data Availability

Custom scripts are shared at GitHub (https://github.com/feifei/scripts_to_share), including the script to scaffold the contigs with optical maps, and the script to update reference genome and annotation based on changes from BWA mapping pileup results. Software including their version information were already listed in the method section.
